# Original Translations

**Published:** 1882-06

**Authors:** 


### Original Translations.

*La France Médicale* contains notes of a case of extremely low temperature following a prolonged exposure to cold during an excess of drunkenness. The patient was picked up unconscious on the street, and died the next day, with a temperature of 79° F. (26° C.) January 27, 1882.

*Prof. M. B. Ball* related before a meeting of the Académie de Médecine, February 21, the case of a patient who had hallucinations of hearing confined to one side, that in which purulent otorrhœa had followed typhoid fever.

*An Accessory Placenta.*— The 19th of February last, a woman gave birth at the Maternité to a living child, head presentation, Fifteen minutes later, the placenta appeared at the vulva, and yet could not be immediately removed. While the midwife was tying a ligature back of the portion expelled, a hæmorrhage took place, and a second placenta was expelled, weighing 40 grams, and the first one 410. The two were formed by a membrane and a few ramifications of blood-vessels.—*Bulletin.*

*A New Stock of Cow-pox.*—Dr. Herviens has lately been experimenting with the virus from a spontaneous case of cow-pox in a heifer at Laforet, near Bordeaux. In relating his experiments before the Académie, the doctor said that he personally believed in the spontaneous origin of cow-pox, and of small-pox also; saying that the first case that ever took place must have been spontaneous, any way ( ? )—*Le Praticien.*

At a meeting of the Société de Chirurgie, February 22, 1882, Mr. Th. Anger reported a small epidemic of tetanus which came under his observation at Cochin Hospital last year. Four patients rapidly died of the disease. While demonstrator of anatomy at Clamart, the same observer witnessed the death of a sloth, together with her six young ones, from tetanus, after they had lived some time in a stable in which two horses had died of the same disease.

*Bone-grafting.*—Mr. MacEwen, of Glasgow, states, in a note to the Institut, that he has succeeded in reproducing om. 114 of the humeral diaphysis by means of six osseous grafts taken from the tibias of children affected with rickety curvatures. These grafts included the periosteum and the marrow also. The new humerus thus formed was half an inch shorter than the opposite one. The grafting was done antiseptically.—*Revue Médicale.*

*A Curious Case of Hysteria, Accompanied with Paraplegia and General Anæsthesia, by Dr. Enrique Gélabert.*—The writer places the following epigraph as a heading to his article: "*Propter solum uterum mulier est id quod est.*" Perhaps these words do not agree with the current scientific idea of our days. However it may be, here is the case: It is that of a girl seventeen years old, who had never menstruated, and had a lymphatic temperament very marked. Four months previous to this, she had entered a shop, in order to strengthen her debilitated constitution, and heal some multilocular adenitis, having remained idle till then. That while her health improved, the adenitis disappeared, and her physiognomy was good. At that time, hysterical accidents suddenly appeared, which the writer referred to *propter solum uterum.* One day, while the girl was following her ordinary duties in the infirmary, seemingly in good health, she suddenly uttered such a loud outcry that the neighbors heard it. The nurse ran to her and interrogated her, whereupon the patient said that she had felt such intense pain in her abdomen that she had not been able to control the cry just uttered. Patient continued at work, but in less than two hours another outcry, more piercing than the first, was heard. This time, the nurse found the patient on the floor, with her face downward, and giving no sign of life. The physician called to see her, two hours later, found the skin cold, the face livid, the pulse small and frequent, the eyesight vague, respiration slow, the senses blunted, and absence of motion. All at once, the patient burst out laughing and singing.

Briefly, in twelve days, she developed a classical attack of hysteria, with bolus, general cutaneous and muscular anæsthesia, which afterward turned into hemianæsthesia, and later into superior hemianæsthesia.

Tartrate of antimony was very efficacious in this case, given hypodermically; besides, let us note that between the fifth and sixth day of the attack, the signs of her first menstruation appeared. The writer explains by that sexual evolution the hysterical attack. The patient did not take any food for eight days.

In relation to this, the writer relates the extraordinary case of a girl who was seized with an hysterical attack in her sleep, and did not take any aliment for seven weeks.—*Revista de Ciencias* in *Revue Médicale.*

*Artificial Feeding in Pulmonary Phthisis.*—Phthisis, says Prof. Peter, is a general and more or less immediate result of tuberculosis; a sort of organic cachexia, whose appearance and progress the physician can, in many cases, delay, even though he is powerless against the cause of the disease, the tubercle.

There is a sinking of the patient, sometimes slow, sometimes rapid, liable to aggravation and improvements, according to the resistance of the constitution. If there is tolerance, the struggle between the constitution and the tubercular products will be longer, and the physician will have time to delay the period at which the tubercles must become phthisical. But the essential factor of such a tolerance lies in the normal functions of the chief systems, especially the digestive.

There is no doubt that insufficient feeding, especially in those hereditarily predisposed, may give rise to tuberculosis. In case the digestive canal is gravely affected and it loses its functional activity, or in cases in which food is not taken in sufficient quantityor quality, tuberculosis may set in. As a proof, there is the frequent co-existence noticed by Lebert, Gallard and Bernutz, of tubercular phthisis with narrowing of the œsophagus, be it cicatricial, cancerous or spasmodic.

Prof. Peter, who was the first to lay stress on the pathogenetic relation between these two pathological processes, relates in his *Leçons de clinique médicale* cases which show plainly this morbid and causative relation: physical impediment to the taking of food; progressive inanition; marasmus; tuberculosis of the lungs. But if insufficient feeding alone is capable, of itself, of giving rise to tuberculous lesions, it must in a greater degree aggravate those already present.

Hence the diet of the phthisical, and of the tuberculous must be the object of special care on the part of the physician. The food must be substantial and variegated. But although it is easy to fill this indication during the early stages of the disease, when the appetite is good, and the digestive canal is not yet attacked, it becomes a difficult matter in ulterior stages, when vomiting and diarrhœa appear, and the appetite diminishes or is succeeded by anorexia.

To remedy the vomiting likely to occur under the circumstances, recourse may be had to washing of the stomach. (See the JOURNAL, April, 1882, p. 393.)

The anorexia remains, which tonics, bitters, mineral waters, etc., are generally insufficient to relieve.

Anorexia is not without its dangers, even in patients possessing sufficient strength, as the hysterical, for instance,—but it is especially dangerous in cachectic patients as the consumptive. These unfortunate patients, consumed by hectic, wasted by profuse sweats, diarrhœas and bronchorrhœas, fatally succumb whenever their appetite fails them.

But, as Mr. Debove wisely remarked, the power of digestion and the appetite, although corresponding in a healthy state may be disassociated in a pathological one; that is, a patient may lack appetite, while still able to digest well. It is also possible in some patients, for the violent disgust accompanying the ingestion of food to excite vomiting.

This way of reasoning, which has been proven practically correct, has been the starting point for artificial alimentation, the results of which have been communicated to the Societé Médicale des Hôpitaux, by its author, Mr. Debove.

The first trial was made on a phthisical patient with advanced signs of pulmonary tuberculosis, and with large caverns. This patient was wasted, had profuse sweats every night, and was so weak as to be hardly able to be up a few hours every day. Every time that he tried to eat, he was seized with disgust, with cough and vomiting. He could not swallow more than one-fourth of a glass of milk.

The failure of general treatment and the indications of an early death called for a new treatment, and Mr. Debove resorted to artificial alimentation.

At a first sitting, a liter, (one quart) of milk was introduced into the stomach, through a sound, after washing of that viscus. It was well borne, and produced neither diarrhœa, vomiting nor nausea. On subsequent days, these injections into the stomach were given twice daily, and the amount of milk increased to two liters, 200 grams (7 ounces) of raw pounded meat, with two eggs.

Notwithstanding that relatively enormous quantity of food, digestion took place, and absorption proceeded in like manner, for the weight of the patient increased about 92 grams a day. However, two slight indigestions took place, one after adding 60 grams of tapioca to the milk, the other following an indiscretion.

With an increase in weight, other results as remarkable appeared. The sweats stopped, sleep returned, the strength increased so that the patient was able to walk a part of the day, the expectoration diminished. But the caverns remained as before.

In a second case, in which the disease was not so far advanced, improvement was more rapid yet, though the washing of the stomach was not practiced. The medium increase in weight was of 192 grains daily. No accident occurred.

Analysis of the urine of this patient showed an increase of urea, a diminishing of the amount of urine, and an increase of albuminuria. This is to be referred to the diet. Some physiologists say that the raw albumen of the egg is partly eliminated through the kidneys, and thus give rise to a slight albuminuria.

The third patient labored under greater disadvantages than either the preceding ones. He had reached the last stage of the disease. A loss of 50 grams per day in weight took place, although the sweats, diarrhœa and insomnia were alleviated.

Mr. Dujardin–Beaumetz has treated fifteen patients by the new process. They increased in weight daily, but not so fast as the two above cases. Mr. Beaumetz gives only half the quantity of food given by Mr. Debove. He also modified the treatment, using a Faucher tube instead of a stiff sound. This tube, which is of an easy introduction, should be at least one centimeter (2-5 inch) in diameter; it should be wet with milk or water before introducing. In case the patient's stomach is very irritable, he injects the aliments immediately, in case it can tolerate previous washing. This is done with a solution of six grams of sodium sulphate in a liter of water.

From the ration proposed by Mr. Debove, he subtracts one egg, and adds, if there is no diarrhœa, one to two hundred grams of cod liver oil to begin with, then some spoonfuls of pepsine, then the food with chloride of sodium.

These results, although not very numerous, are encouraging enough to incite to new trials. Forced alimentation seems to offer important advantages which recommend it to practitioners. It can be applied to the last stage of phthisis, which was formerly left without available treatment. This method accordingly constitutes a precious means of treatment and marks a real progress. However, it is not devoid of all risks.

Thomas M. Denos saw, in a case of phthisis in its last stage, access of suffocation and vomiting, with reflux of the milk through the nostrils, and its introduction into the trachea, soon following on the forced ingestion of a liter of milk. Patient died of pneumonia in less than two days.

Notwithstanding this accident, Mr. Denos declares in favor of the forced alimentation of phthisical patients; recommending at the same time to proceed with prudence and gently, and introduce smaller doses in succession. He does not advise this treatment during febrile accesses. These advices should be carefully noted down.—F. LEPRÉVOST, in *Revue Médicale*, Feb. 4, 1882.

